# Establishing Antibacterial Multilayer Films on the Surface of Direct Metal Laser Sintered Titanium Primed with Phase-Transited Lysozyme

**DOI:** 10.1038/srep36408

**Published:** 2016-11-08

**Authors:** Binbin Guan, Haorong Wang, Ruiqing Xu, Guoying Zheng, Jie Yang, Zihao Liu, Man Cao, Mingyao Wu, Jinhua Song, Neng Li, Ting Li, Qing Cai, Xiaoping Yang, Yanqiu Li, Xu Zhang

**Affiliations:** 1 The School and Hospital of Stomatology, Tianjin Medical University, 12 Observatory Road, Tianjin 300070, PR China; 2 NanKaiHospital, 122 Three Weft Road, Tianjin, 300110, PR China; 3 Beijing Laboratory of Biomedical Materials, College of Materials Science and Engineering, Beijing University of Chemical Technology, Beijing 100029, PR China; 4 Beijing Institute of Aeronautical Materials, Beijing 100089, PR China; 5 Bybo Dental Group, 18th Building 4, Qinian Main Street, Beijing 100010, PR China

## Abstract

Direct metal laser sintering is a technology that allows the fabrication of titanium (Ti) implants with a functional gradation of porosity and surface roughness according to three-dimensional (3D) computer data. The surface roughness of direct metal laser sintered titanium (DMLS-Ti) implants may provide abundant binding sites for bacteria. Bacterial colonization and subsequent biofilm formation can cause unsatisfactory cell adhesion and implant-related infections. To prevent such infections, a novel phase-transited lysozyme (PTL) was utilized as an initial functional layer to simply and effectively prime DMLS-Ti surfaces for subsequent coating with antibacterial multilayers. The purpose of the present study was to establish a surface with dual biological functionality. The minocycline-loaded polyelectrolyte multilayers of hyaluronic acid (HA) and chitosan (CS) formed via a layer-by-layer (LbL) self-assembly technique on PTL-functionalized DMLS-Ti were designed to inhibit pathogenic microbial infections while allowing the DMLS-Ti itself and the modified coatings to retain acceptable biocompatibility. The experimental results indicate that the DMLS-Ti and the hydrogel treated surfaces can inhibit early bacterial adhesion while completely preserving osteoblast functions. This design is expected to gain considerable interest in the medical field and to have good potential for applications in multifunctional DMLS-Ti implants.

Titanium (Ti) and its alloys are commonly used in the orthopaedic and dental implant fields owing to their desirable biocompatibility and excellent physicochemical properties^1^,^2^. However, within the complex oral microbiomes, implant-associated infections, including peri-implant mucositis and peri-implantitis, increase the probability of implant failure and may eventually result in integrated implant loosening and detachment^3^,^4^. Bacterial colonization and subsequent biofilm formation are primary causes of implant-associated infections. Although various measures, such as disinfection and stringent aseptic surgical protocols, have been proposed to eliminate bacterial contamination in surgery, bacterial colonization will still occur after installation^5^,^6^. *Streptococcus gordonii* (*S. gordonii*) and *Streptococcus sanguis* (*S. sanguis*) are considered the predominant microorganisms of early colonization, as they prepare more surface anchor sites as well as suitable growth conditions for late colonizers, resulting in the formation of a complex dental biofilm^7^,^8^. Once a biofilm is formed, it is difficult to remove, as the biofilm protects the bacterial colonies from host defence systems and from bactericidal agents^9^,^10^. When this occurs, the final outcome is often implant removal. Therefore, the most effective method of prevention is to establish antibacterial coatings on the surfaces of dental implants to inhibit early bacterial colonization during the process of biofilm formation.

Currently, direct metal laser sintering is one of the most commonly used metal-based three-dimensional (3D) printing techniques. This technology is capable of fabricating a functional gradation of globular protrusions, interconnected pores, and irregular crevices, as well as a rough surface across an implant^11^. Specific porous and rough surface microstructures form from metal powder that is not completely sintered. The porosity of a direct metal laser-sintered Ti (DMLS-Ti) implant could be used to modulate its mechanical properties such that the elastic modulus of the implant mimics that of the host bone^11^,^12^,^13^. Such structures can effectively minimize shielding effects that are associated with severe bone resorption and septic loosening^14^. Meanwhile, the implant-bone response is considered to be associated with implant surface topography^15^. The surface roughness of DMLS-Ti implants increases the potential for cell attachment and proliferation, as well as the production of bone morphogenetic proteins, vascular endothelial growth factor and bone-specific proteins^16^,^17^, which are essential factors for enhancing osseointegration. However, at the same time, a clear positive correlation has been found between implant surface roughness and bacterial adhesion and aggregation^18^. Generally, surface roughness provides more favourable sites for bacterial colonization. Although the morphology of DMLS-Ti implants is a promising alternative to the topographies of conventional implant surfaces, until now, little attention has been focused on whether DMLS-Ti implants with rough surfaces are associated with an increased risk of bacterial adhesion. Moreover, within the unique, dynamic tissue environment around an implant, microbial biofilm growth on the implant surface likely competes with tissue-biomaterial integration^19^,^20^. If bacteria predominate, an implant surface will be rapidly covered by a biofilm, if tissue cells predominate, then the surface will be covered by normal tissue^20^. For this reason, it is necessary to establish antibacterial coatings for DMLS-Ti implants to prevent biofilm formation and allow tissue cell growth.

The process of implant-associated infection is comparable to that of advanced periodontitis. Antibacterial drugs have been paid an increasing amount of attention for the clinical treatment of periodontal diseases. In particular, minocycline hydrochloride (MH), or its ointment, is an efficient, semi-synthetic tetracycline, which has become routinely prescribed because of its broad antimicrobial spectrum, potency against bacteria, inhibition of collagenase metabolism, and promotion of periodontal tissue regeneration^21^,^22^. However, considering the specificity of the treatment conditions of periodontal tissue after implant installation, maintaining long-term antibacterial activity on dental implants remains a challenge. Layer-by-layer (LbL) self-assembly method produces excellent laminated structures with semi-permeable and perm-selective properties, which can be used to control and guide drug delivery^23^,^24^,^25^. Among all the polymers available for used in drug delivery systems, degradable materials are highly recommended. Multilayers fabricated by LbL self-assembly and composed of negatively charged hyaluronic acid (HA) and positively charged chitosan (CS) have been extensively investigated for applications in drug delivery systems^23^,^26^. HA and CS are natural polysaccharides with nontoxic, biodegradable, biocompatible, and film-forming properties^27^,^28^. More importantly, CS films are expected to be able to encapsulate MH and thus achieve better controlled-release effects^29^. Accordingly, in this study, we chose HA/CS polyelectrolyte multilayers to achieve a sustained rate of MH release into inflamed lesion sites and prevent the formation of bacterial biofilms.

To effectively and firmly combine the antibacterial multilayers coating with the implant surface, it is crucial to pretreat the implant surfaces to constructs an excellent activated surface. Chemical methods are usually considered the most efficient ways to modify an implant surface via cross-linking agents, such as glutaraldehyde, which could compromise the biocompatibility of the multilayers coating^30^. These methods increase the possibility of additional chemical effects on host tissue due to the involvement of multiple chemical activation steps, complex chemical groups, and long processing times. Consequently, there is an urgent need to develop simple, nontoxic and effective methods of priming implant surfaces for modification. With the development of biomaterial surface modifications, dopamine, or poly-dopamine, was found to adhere to almost any solid surface without requiring a surface pretreatment^31^,^32^. However, these modifications still have some undesirable effects, such as the tendency for surface discolouration due to oxidations of the metal^33^. In this study, a novel phase-transited lysozyme (PTL) was implemented instead of dopamine or poly-dopamine for surface functionalization. This strategy provides an approach for establishing modified surfaces with less discolouration, fewer chemical steps, greener processing, less expensive ingredients and controlled reversibility. The phase-transition process is based on the β-sheet transition found in lysozyme microfibres, which exhibit reliable “superglue-like” adhesion such that they can be stably and quickly attached onto a variety of substrate surfaces, regardless of the substrate type^34^,^35^. The modified surface becomes occupied by a network of interconnected microfibres, each with a diameter of 0.5–1 μm, thereby providing a lysozyme coating with a thickness that can be adjusted to range from 30–230 nm^34^. Moreover, the PTL confers not only moderate hydrophilicity but also positive charges^34^, and thus combines with negatively charged HA through straightforward electrostatic interactions. Following this process, three bilayers of an HA/CS coating can be obtained on a DMLS-Ti surface primed with PTL.

Bacterial adhesion and biofilm formation on a Ti surface can vary as they are dependent on microbial species and surface texture^36^. Few studies have attempted to understand the effects of bacterial adhesion on the specific surface of DMLS-Ti implants. This study aimed to design and establish polyelectrolyte multilayers of minocycline-loaded HA/CS via LbL self-assembly on the surface of DMLS-Ti implants pre-treated with PTL. Notably, this is the first attempt to load such an antimicrobial coating onto DMLS-Ti implants. However, the deposition of the PTL coating and HA/CS hydrogel may change the mechanical properties of the material interface. Based on the above-mentioned factors, the correlation between the mechanical characteristics of the material interface and the behaviour of the bacterial and mammalian cells need to be evaluated. We hypothesized that DMLS-Ti surfaces with an initial layer of a novel PTL and an additional minocycline-loaded, the multilayers coating would exhibit not only efficient and sustainable antibacterial efficacy but also excellent biocompatibility.

## Results

### Surface characterization

**Figure 1:**
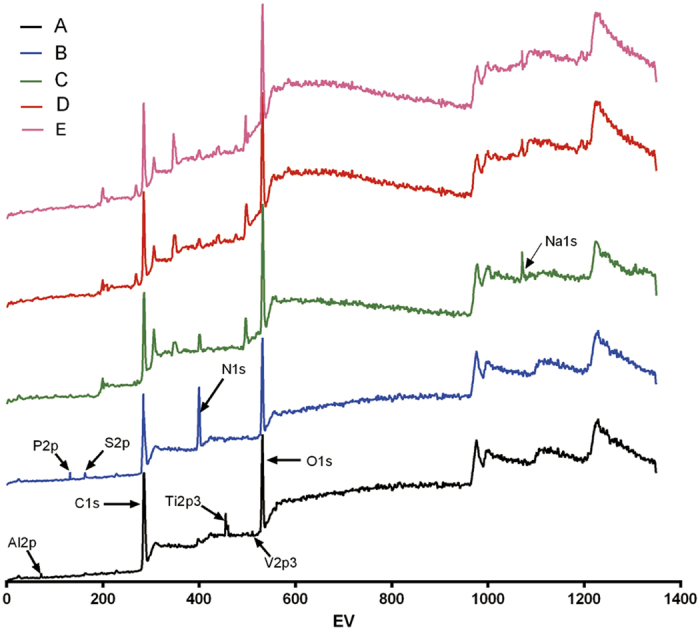
XPS wide-scan spectra of (**A**) DMLS-Ti, (**B**) PTL-primed DMLS-Ti, (**C**) PTL-Ti-HA, (**D**) PTL-Ti-HA/CS(MH), and (**E**) PTL-Ti-[HA/CS(MH)]_3_ after having been immersed in deionized water for 7 days.

Surface chemical compositions were determined by X-ray photoelectron spectroscopy (XPS), as shown in Fig. 1 and Table 1; the results illustrated the different stages of surface functionalization on the DMLS-Ti and modified Ti discs. All binding energies were referenced to the C 1s spectrum peak (284.8 eV) as an internal reference after calibrating peak positions. The spectrum of the DMLS-Ti (Fig. 1A) showed that the main components included C 1s, Ti 2p3 (458.4 eV), Al 2p (74.12 eV), V 2p3 (512.3 eV), O 1s (530 eV) and N 1s (402 eV). The appearance of S 2p (165 eV) and P 2p (133 eV) peaks, derived from the tris (2-carboxyethyl)phosphine (TCEP), and the disappearance of the Ti 2p3, Al 2p, and V 2p3 peaks on the modified DMLS-Ti surface indicated that the PTL had been successfully immobilized (Fig. 1B). This result was also confirmed by the quantitative XPS data (Table 1). The spectrum peak of Na 1s (1072 eV), originating from the hyaluronate, indicated that the HA had been successfully deposited on the PTL-primed DMLS-Ti surface (Fig. 1C and Table 1). As the number of layers increased from the addition of HA/CS(MH), the apparent increase in the atomic N ratio provided evidence that the MH had successfully been deposited on the Ti substrate (Table 1).

**Figure 2:**
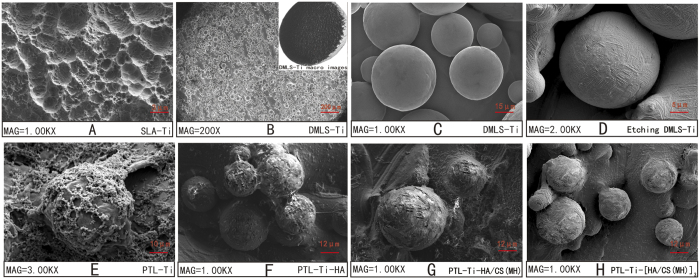
SEM images showing the surfaces of (**A**) SLA Ti, (**B**,**C**) DMLS-Ti, (**D**) etched DMLS-Ti, (**E**) PTL-Ti, (**F**) PTL-Ti-HA, (**G**) PTL-Ti-HA/CS(MH) and (**H**) PTL-Ti-[HA/CS(MH)]_3_. One macro image from a manufactured DMLS-Ti specimen is shown in an insert in Fig. 2B.

**Figure 3:**
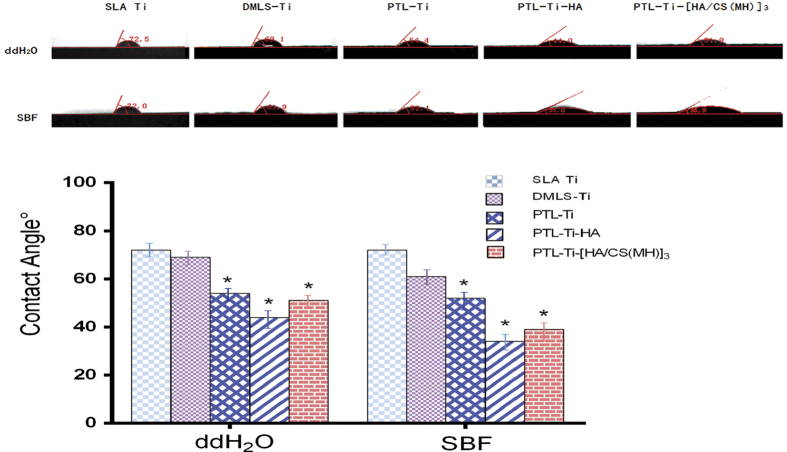
Contact angle of water and SBF on the surface of the tested samples (n=3). *P < 0.05, compared with SLA Ti.

The scanning electron microscopy (SEM) images shown in Fig. 2A–H illustrate the morphology of an SLA Ti disc, a DMLS-Ti disc, an etched DMLS-Ti disc, a PTL-Ti disc, a PTL-Ti-HA disc and a PTL-Ti-[HA/CS(MH)]_3_ disc, respectively. One macro image from a manufactured DMLS-Ti specimen is shown in an insert in Fig. 2B. These results showed that the DMLS-Ti discs had a topography consisting of spherical micro-islands, micro-holes and micro-channels. The diameter of the spherical features ranged from 15–50 μm, with an average of 25 μm. The results (Fig. 2E) also showed that the PTL had a diameter of 0.5–1 μm and formed a necklace-like microfibre network attached stably to the DMLS-Ti disc. The final SEM images (Fig. 2G,H) showed that the multilayers of HA and CS loaded with minocycline covered the rough surface of the DMLS-Ti disc after self-assembly. The static contact angle measurements for water and simulated body fluid (SBF) showed that the DMLS-Ti and modified Ti discs were more hydrophilic than the SLA Ti discs (Fig. 3). The water contact angle on the different stages of the LbL self-assembled multilayers decreased sharply from 72.5° ± 1.5° to 51.9° ± 1.3°. Meanwhile, the SBF contact angles showed similar trends and were generally lower than the water contact angles.

### Mechanical properties

**Figure 4:**
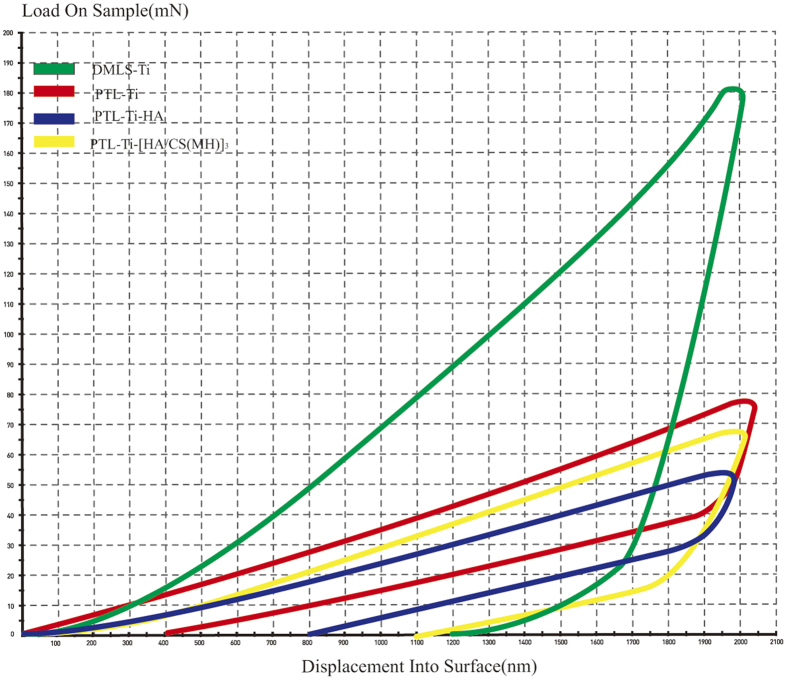
Nano-indentation data showing the stiffness and Young’s modulus of the DMLS-Ti and the multilayer films. The hardness was derived by dividing the load by the area of contact. The slope of the unloading curve provided a measure of the elastic modulus.

Nano-indentation was used to assess the hardness and Young’s modulus of the various functionalized layers on the DMLS-Ti samples. The load-displacement curves are shown in Fig. 4. The hardness and modulus of DMLS-Ti were found to be 2.1 ± 0.4 and 71.2 ±  5.2 GPa (Table 2), respectively. After the surface modification, a decrease in both the hardness and Young’s modulus was observed on the DMLS-Ti surface primed with PTL and coated with self-assembled multilayers.

### Release of minocycline

**Figure 5:**
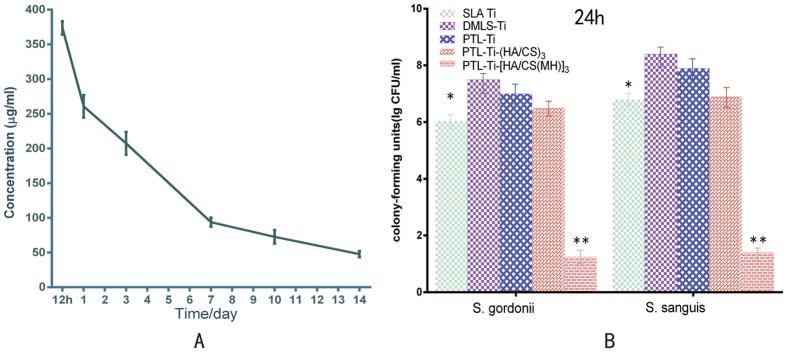
(**A**) Release curve of minocycline from the PTL-Ti-[HA/CS(MH)]_3_ after 1, 3, 7, 10 and 14 days in PBS. (n = 3). (**B**) Characterization of the antibacterial efficacy against *S. gordonii* and *S. sanguis* as assessed by CFU counting after 24 h (n = 3). *P < 0.05 and **P < 0.01, compared with DMLS-Ti.

The release curve of minocycline from the self-assembled multilayers is shown in Fig. 5A. The release of minocycline from the LbL film was steady, except for an initial burst release within 24 h, and gradually stabilized after 7 days. The final average concentration of released minocycline was 43 ± 4.1 μg/ml.

### Inhibition of biofilm formation

**Figure 6:**
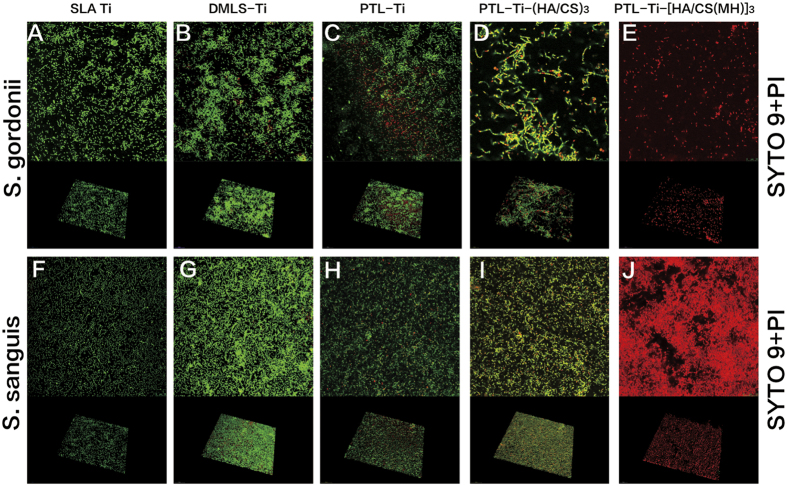
CLSM 2D and 3D images of DMLS-Ti and functionalized DMLS-Ti discs cultured with *S. gordonii* and *S. sanguis*. Images of live cells stained with SYTO 9 (green) and dead cells stained with propidium iodide (red). (**A**,**F**) SLA Ti. (**B**,**G**) DMLS-Ti. (**C**,**H**) PTL-Ti. (**D**,**I**) PTL-Ti-(HA/CS)_3_. (**E**,**J**) PTL-Ti-[HA/CS(MH)]_3_.

The antimicrobial efficacy of the self-assembled multilayers against the oral bacteria *S. gordonii* and *S. sanguis*, two crucial pathogenic species in the biofilm formation process, were evaluated by the spread plate method and confocal laser scanning microscopy (CLSM). Specifically, the CLSM observations showed that more live bacteria adhered to the DMLS-Ti samples (Fig. 6B,G) than to the SLA Ti samples (Fig. 6A,F). The quantitative results (Fig. 5B) showed that the concentrations of bacteria on the DMLS-Ti surfaces (*S. gordonii*, 3.2 ± 0.2 × 10^7^ colony-forming units (CFU)/ml and *S. sanguis*, 2.5 ± 0.1 × 10^8^ CFU/ml) were much higher than those on the SLA Ti surfaces (*S. gordonii*,1.1 ± 0.2 × 10^6^ CFU/ml and *S. sanguis*, 6.3 ± 0.2 × 10^6^ CFU/ml). As shown in Fig. 6C,H, a decrease in the percentage of viable bacteria and partially red-stained bacteria was observed on the PTL-primed DMLS-Ti surface. Meanwhile, the PTL-Ti-(HA/CS)_3_ samples also exhibited lower antibacterial efficacy (*S. gordonii*, 3.2 ± 0.2 × 10^6^ CFU/ml and *S. sanguis*, 7.9 ± 0.2 × 10^6^ CFU/ml). In addition, compared with the other groups, the surfaces coated with the antibacterial self-assembled multilayers showed significantly fewer bacterial colonies (*S. gordonii*, 17.8 ± 0.2 CFU/ml and *S. sanguis*, 25.1 ± 0.3 CFU/ml). These findings are consistent with the CLSM observations. The reconstructed 3D images shown in Fig. 6 further support the results from the CLSM images. Therefore, the multilayer films fabricated by LbL self-assembly on the surface of DMLS-Ti exhibited satisfactory antimicrobial properties which would inhibit bacterial biofilm formation.

### Cytotoxicity

**Figure 7:**
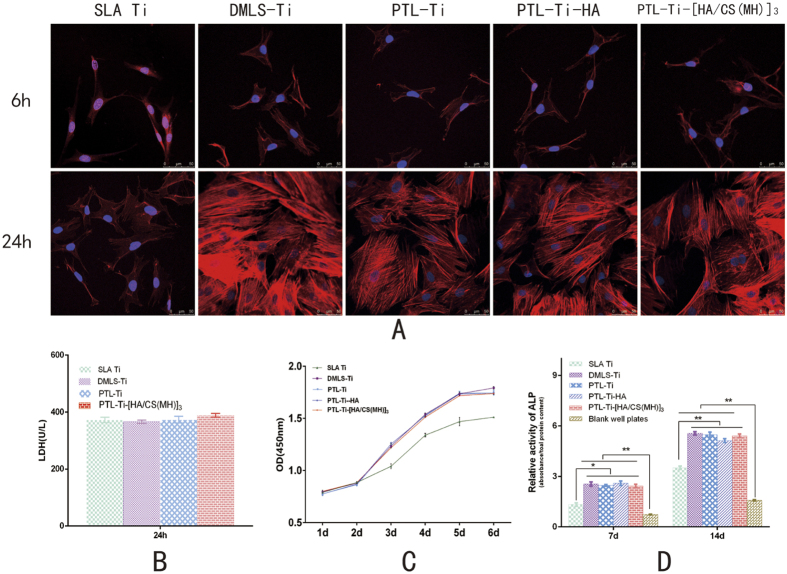
(**A**) CLSM images of MC3T3- E1 cells attached on the DMLS-Ti, PTL-Ti, PTL-Ti-HA, PTL-Ti-[HA-CS(MH)]_3_ and SLA Ti surfaces after 6 and 24 h of culture. (**B**) LDH activity after culturing cells on the specimens for 24 h. (**C**) Growth curves of MC3T3-E1 cells on DMLS-Ti, PTL-Ti, PTL-Ti-HA, PTL-Ti-[HA-CS(MH)]_3_ and SLA Ti surfaces. (**D**) ALP activity of MC3T3-E1 cells cultured on the specimens for 7 and 14 days. The data are expressed as the mean ± standard deviation (n = 3). *P < 0.05 and **P < 0.01 represent levels of significance.

A cytotoxicity assay (Fig. 7B) was performed by evaluating the lactate dehydrogenase (LDH) activity of MC3T3-E1 cells after 24 h of culture on PTL-primed DMLS-Ti and PTL-Ti-[HA/CS(MH)]_3_ samples. The samples did not show an obvious cytotoxicity compared to the SLA Ti and DMLS-Ti control samples. No significant increases in LDH activity were observed in any group at any time point.

### MC3T3-E1 cell adhesion and proliferation

MC3T3-E1 cell adhesion was assayed by staining with phalloidin and DAPI to visualize the F-actin (red) and the nuclei (blue), as shown in Fig. 7A. After 6 h of culture, the F-actin had extended into a larger polygonal morphology on the DMLS-Ti surfaces, while spindle cells could be observed on the SLA Ti surfaces. After culturing for 24 h, no significant differences in the number of adherent cells were found among the tested surfaces. Although pseudopodia were extended on the SLA Ti surfaces, they were obviously shorter than those on the DMLS-Ti surfaces.

Regarding MC3T3-E1 cell proliferation, a logarithmic growth proliferation curve (Fig. 7C) was found for each sample surface after one week. There were no apparent differences among the five groups in the first two days. Subsequently, the SLA Ti samples exhibited slightly lower cell viability than did the other groups. On the fifth day, the proliferation on the SLA Ti samples reached a plateau. However, the cells on the DMLS-Ti, PTL-Ti, PTL-Ti-HA and PTL-Ti-[HA/CS(MH)]_3_ samples continued to grow, reaching a plateau after 5 days of culture. In addition, compared with the DMLS samples, the PTL-Ti-[HA/CS(MH)]_3_ samples did not show a significant difference in cell viability (P > 0.05). As a whole, the five groups showed similar trends in MC3T3-E1 cell proliferation.

### Alkaline phosphatase (ALP) activity

Figure 7D shows the ALP activity in cell lysates after 7 and 14 days. ALP, a marker of early osteoblastic differentiation, was elevated in cells cultured on samples compared with cells cultured in blank wells. ALP activity was moderately different between SLA and DMLS samples on the 7th day (P < 0.05). By the 14th day, the ALP activity in the DMLS groups was significantly higher, demonstrating an osteogenic advantage (P < 0.01). Meanwhile, no apparent differences in ALP activity were found between the DMLS-Ti and modified surfaces after 7 and 14 days.

## Discussion

Studies have paid enormous attention to the reliability of DMLS-Ti dental implants, including their biological, clinical, histological, and mechanical properties^37^. The correlations between bacterial adhesion and the specific surface properties of DMLS-Ti have not yet been studied. This study indicated that bacteria are more likely to colonize rough DMLS-Ti surfaces. In contrast, some results have suggested that there is no relationship between surface roughness and the capacity for biofilm formation^38^. This type of discrepancy may be due to the use of different bacterial strains, culture conditions, or other environmental factors in these different studies. The selection of surface modification methods and antimicrobial coatings are the main challenges of retaining the DMLS-Ti surface characteristics while minimizing the influences on osteoblast functions. This study confirmed the successful deposition of an HA/CS multilayers coating via LbL technology on the surface of DMLS-Ti primed with PTL and the subsequent inhibition of biofilm formation.

In the present work, to acquire a multifunctional DMLS-Ti surface, acid etching and a newly discovered PTL were implemented. The purpose of the additional acid-etching process is to increase the surface microroughness and remove weakly bound metal particles^17^. Metal particles that are not completely sintered could potentially be detached during or after implant insertion. Moreover, the metallic powder may enter or remain in surrounding tissues, potentially disturbing the immune system. The SEM images showed that the average spherical particle diameter was 25 μm, which is similar to the findings of previous research showing that DMLS-Ti surfaces were covered by microspheres 15.8 ± 7.1 μm in diameter^11^. These results indicated the maturity of the DMLS-Ti manufacturing technology. PTL is a very strong initial layer that sets a foundation for the subsequent self-assembly of multilayers. It has been suggested that this binding could endure extensive ultrasonic washing and maintain its integrity when exposed to other harsh reagents but selectively disintegrate in guanidine solution. The mechanism of PTL adhesion is a complex and comprehensive response process. In brief, the disulphide bonds of the lysozyme are broken by TCEP, a phase transition process occurs, and the internal lysozyme forms a β-sheet structure. This structure is similar to that of amyloid, which is a multi-specific adhesive protein^34^. The SEM images (Fig. 2E) and XPS data provide evidence supporting the deposition of PTL on the surface of DMLS-Ti.

Ideally, antibiotic-loaded implant coatings should have a release profile corresponding with the wound-healing process (10–14 days). In this study, we found that the release of minocycline from the self-assembled multilayers continued throughout the period of normal wound healing. The release of minocycline from the self-assembled multilayers could be grossly divided into three phases. Because some antibacterial agents were attached to the outermost layer, there was an initial burst release of minocycline from the antibacterial multilayers coating within the first 24 h. This burst could effectively kill microorganisms introduced during the peri-operative or early post-operative phases of implant placement. Then, the concentration of released minocycline decreased gradually in the intermediate phase, indicating the role of the multilayers in the controlled released. Finally, the concentration of released minocycline decreased slowly along with the decomposition of the antibacterial multilayers coating. In addition, previous research has shown that the minimum inhibitory concentration (MIC) of minocycline (5 μg/ml) has been proposed to inhibit 98% of subgingival bacterial species^39^. It is worth noting that the concentration of released minocycline during the final phase was still higher than the MIC. Accordingly, the multilayers coating designed for DMLS-Ti surfaces and constructed by LbL self-assembly technique exhibited the expected enhanced loading capacity and controlled release of minocycline. However, to prevent the occurrence of new antibiotic-resistant bacteria, the release of the antibiotics should cease after a wound has healed^40^. Thus, optimal minocycline loading and release profiles should be further investigated through animal models in the future.

Early bacterial adhesion to an implant surface is believed to be a critical pathogenic component of such infections; thus, an important strategy is to inhibit early bacterial adhesion. As *S. gordonii* and *S. sanguis* serve as anchors that enable and promote the subsequent attachment of other species^5^,^7^, they were selected for use in the antimicrobial assays in our study. The results showed that both *S. gordonii* and *S. sanguis* showed more scattered populations on DMLS-Ti surfaces. These are the first results to indicate that the rough DMLS-Ti surface increases early microorganism adhesion and colonization (Fig. 6B,G). Interestingly, the CLSM images (Fig. 6C,H) showed that the PTL-Ti samples were partially stained red, indicating that PTL exhibits relatively weak antibacterial activity. In a previous study, Zhong *et al*.^26^ also found red-stained, dead *S. aureus* cells on the surfaces of PTL-primed Ti. In that study, the relatively strong antibacterial activity may be a result of the use of different bacteria. The SEM images showed that the DMLS-Ti surface occupied by a network of interconnected microfibres, each with a diameter of sub-micronized (0.5–1 μm) pattern, the length-scale-mediated differential interactions may affect cell behaviour. Denatured lysozyme can kill bacteria by a non-enzymatic pathway, allowing the antimicrobial function to remain preserved^41^; however, the exact mechanisms are not clearly understood and require further investigation. Fortunately, consistent with our expectations, *S. gordonii* and *S. sanguis* adhering to PTL-Ti-[HA/CS(MH)]_3_ were completely stained red (Fig. 6E,J), indicating that the minocycline-loaded, multilayers coating fabricated via LbL self-assembly possessed a high antimicrobial efficacy. The results of the CFU counting assays results (Fig. 5B) were consistent with the CLSM results (Fig. 6). As indicated by the of PTL-Ti-(HA/CS)_3_ groups (Fig. 6D,I), we can conclude that the significant biofilm inhibition was due to the amount of MH loaded rather than the hydrogel property. In summary, PTL-Ti-[HA/CS(MH)]_3_ exhibited a strong antibacterial effect against the early colonizers, thus inhibiting subsequent biofilm formation.

Although the multilayers coating showed promising antibacterial properties, it is necessary to evaluate its biocompatibility for potential applications. Considering the biodegradability of the multilayers coating, the cytotoxicity of the final layer was tested. Studies have demonstrated that multilayers of CS and HA are nontoxic and biocompatible^24^. The results of this study indicated that the DMLS-Ti surface primed with PTL and PTL-Ti-[HA/CS(MH)]_3_ were not cytotoxic (Fig. 7B). Regarding osteoblast functions on DMLS-Ti and PTL-Ti-[HA/CS(MH)]_3_, our study indicated that DMLS-Ti had a cytocompatibility similar to that of SLA Ti. We found almost no differences in the adhesion and proliferation of MC3T3-E1 cells on DMLS-Ti discs compared with SLA Ti surfaces (P > 0.05). The CLSM images of the F-actin and cell nuclei showed both the morphology and number of the early adherent cells. However, on the surfaces of the SLA Ti samples, the pseudopodia were obviously shorter than those on the surfaces of the DMLS-Ti samples. Likewise, Mangano *et al*. found that cells were irregularly shaped and spanned across intervening crevices by means of extended pseudopodia^17^. The cytoskeleton plays a very important role in cell behaviour, and the cytoskeletal changes indicated that the MC3T3-E1 cells tended to migrate on the DMLS-Ti surfaces. Moreover, significant differences were found in early osteogenesis-related ALP activity in the cell lysates, with ALP activity that would clearly be advantageous for osteogenesis being observed in the DMLS-Ti group (Fig. 7D). In another study, Mangano *et al*.^16^ demonstrated that dental pulp stem cells could quickly differentiate into osteoblasts and endotheliocytes; meanwhile, in this study, improved osteoblast differentiation and bone morphogenetic protein production occurred more rapidly on the DMLS-Ti surface than on the other surfaces. This finding indicates that compared with the SLA Ti surfaces, the DMLS-Ti surfaces have better osteogenic capacity.

Bacteria and mammalian cells can perceive mechanical signals and transduce these signals into corresponding biological signals, thereby regulating cell adhesion, spreading, proliferation and differentiation^42^. Generally, cells prefer to grow on stiff substrates such as tissue culture polystyrene or glass. The nano-indentation results suggested a decrease in the stiffness and Young’s modulus after the deposition of the PTL and HA/CS multilayers coating. However, it is worth mentioning that the cellular effects of PTL-Ti, PTL-Ti-HA, and PTL-Ti-[HA/CS(MH)]_3_ were similar to those of DMLS-Ti and that the influence of the hydrogel layer on the cellular and bacterial behaviour was weak. Chua *et al*.^23^ suggested that HA/CS polyelectrolyte multilayer functionalized Ti has little efficacy in terms of osteoblast adhesion. Accordingly, the interactions between the DMLS-Ti structure and the multilayers coating may exert a mutual influences on the cells; the effect of the DMLS-Ti structure on the multilayers coating may be the main reason for these results. The SEM images clearly showed visible, spherical micro-islands after self-assembly (Fig. 2G,H). Polyelectrolyte multilayer films can be considered two-dimensional (2D) materials. Although they cannot form sufficiently porous 3D scaffold structures, they can be deposited on porous materials to provide more space for cell growth^43^. Compared with the original spherical morphology of the DMLS-Ti surface, the coating comprising three bilayers did not change the overall appearance. Therefore, DMLS-Ti surfaces coated with the polyelectrolyte multilayer presented in this study were demonstrated to be biocompatible.

## Conclusion

This present study demonstrated the feasibility of loading minocycline into HA/CS polyelectrolyte multilayers on DMLS-Ti surfaces primed with PTL to achieve antibacterial efficacy while retaining osteoblast functions. To effectively activate the DMLS-Ti surface, the results of this study suggest that the novel use of PTL for implant surface modification is a promising priming method due to its advantages of simple design, low cost, high safety and easy accessibility. At the same time, LbL self-assembly technology is also applicable to this specific morphology of DMLS-Ti. There is no doubt that the combination of direct metal laser sintering technology and subsequent antibacterial functionalization will gain considerable interest in the medical field because of its infinite potential for producing multifunctional Ti implants.

## Methods

### Materials

Ti disc specimens were divided into two groups, a DMLS group (experimental group), and an SLA group (control group). Each Ti disc (Ti–6Al–4 V) was Ф 10 mm × 1 mm in size. All Ti discs were obtained from the Beijing Institute of Aeronautical Materials. The experimental Ti discs were fabricated by direct metal laser sintering technology. A master alloy powder (Ti–6Al–4V) with a particle size of 25–45 μm μm was used as the basic material. Manufacture of DMLS-Ti was manufactured in an argon atmosphere using a powerful Yb (Ytterbium) fibre laser system (EOS M280, Germany), a wavelength of 1054 nm, a continuous power of 200 W, a laser scanning speed of 7 m/s, and a laser spot size of 0.1 mm. CS was purchased from Fluka; the molecular mass was 400,000 Da, and the degree of deacetylation was 82%. Sodium hyaluronate (1 g) and MH were purchased from Sangon Co., Ltd. (Shanghai, China). Lysozyme, HEPES buffer (pH 7.4 10 mM) and TCEP were purchased from Sigma-Aldrich. SBF was purchased from MACGENE (Beijing, China). *S. gordonii* and *S. sanguis* were obtained from the China General Microbiological Culture Collection Centre. MC3T3-E1 murine pre-osteoblasts were obtained from the Type Culture Collection of the Chinese Academy of Sciences (Shanghai, China).

### Specimen preparation

To remove residual impurities remaining after the fabricating process, the specimens were immersed in NaOH (20 g/l) and hydrogen peroxide (20 g/l) at 80 °C for 30 min and then ultrasonically washed with acetone, anhydrous ethanol and distilled water for 15 min each. Subsequently, the DMLS-Ti specimens were exposed to 40% hydrofluoric acid (HF) vapour at room temperature for 5 min. Finally, all the Ti specimens were cleaned ultrasonically in double-distilled water (ddH_2_O) for 30 min and sterilized in an autoclave at 120 °C for 30 min prior to the *in vitro* experiments.

To functionalize the DMLS-Ti surface with a PTL coating, the samples were first dipped into a lysozyme (2 mg/ml) HEPES solution (10 mM) mixed with a TCEP (50 mM, pH 7.2) HEPES solution (1:1 v/v ratio) and were then incubated at room temperature and 80% humidity for 2 h. Subsequently, the Ti discs were removed from solution and washed extensively with HEPES solution to eliminate non-adherent material.

### Preparation of multilayer films

**Figure 8:**
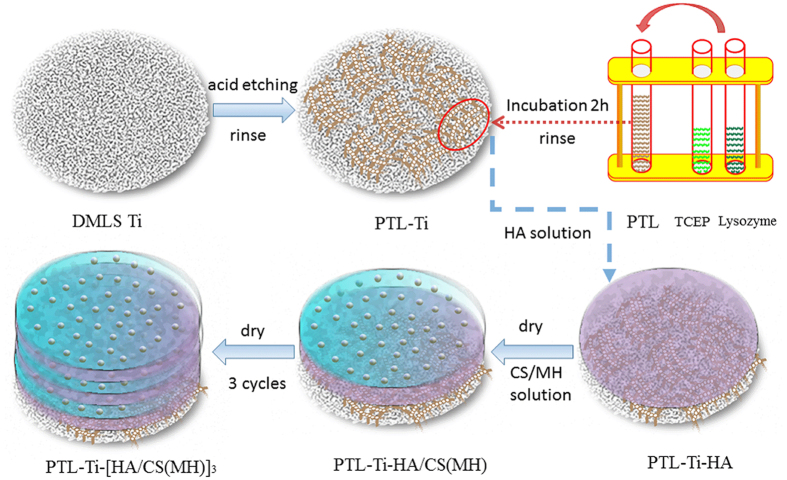
Schematic diagram showing the process of fabricating minocycline-loaded HA/CS multilayers coating via LbL self-assembly on the surfaces of PTL-primed DMLS-Ti substrates.

To deposit a uniform multilayer coating on the surface of the PTL-primed Ti samples, the DMLS-Ti discs were dipped in each of the polyelectrolyte solutions for 15 min. First, the samples were dipped in an anionic HA solution (1 mg/ml in 0.14 M aqueous NaCl, pH 6.0). Then, the samples were immersed in a cationic CS(MH) solution under stirring at 1500 r/min for 30 min (1.5 mg/ml CS in 0.1 M HAc containing 0.14 M NaCl and 2% (w/v) minocycline, pH 5.0). Each dip was followed by washing with 0.14 M NaCl for 5 min and drying with nitrogen gas. The multilayers were obtained by repeating the cycle three times. PTL-Ti, PTL-Ti-HA, PTL-Ti-HA/CS(MH), and PTL-Ti-[HA/CS(MH)]_3_ were used to denote each DMLS-Ti implant dip step, respectively. Finally, these functionalized Ti discs were stored at 80% relative humidity until the subsequent experiments. The whole process is shown in Fig. 8.

### Surface characterization

After being immersed in deionized water for 7 days and then dried, uncoated DMLS-Ti samples and DMLS-Ti samples of each film layer were analysed by XPS on an AXIS His spectrometer (Kratos Analytical Ltd., UK) to identify the chemical compositions. The contact angle of each layer with ddH_2_O and SBF was measured using a drop-shape analysis system (JC2000D1, Micaren, China) at room temperature. The mean static contact angle of each Ti surface was determined from measurements performed in triplicate.

The surface microstructure morphology of the SLA Ti, DMLS-Ti and the multilayer films was evaluated field-emission SEM (SUPRA 55 SAPPHIRE, Germany) with an energy-dispersive X-ray spectrometer (EDX, Germany).

The stiffness and Young’s modulus of the DMLS-Ti and the multilayer films were determined via a nano-indentation analysis. To increase measurement reliability, the highest central point was selected for the loading-unloading sequence in the experiment. The load and displacement of the indenter were continuously monitored. The hardness was derived by dividing the load by the area of contact. The slope of the unloading curve provided a measure of the elastic modulus.

### *In vitro* release profile of minocycline from the composite

To analyse the release profile of minocycline, multilayer film-coated DMLS-Ti samples were immersed in a phosphate-buffered saline (PBS) solution (pH 7.4) in a 48-well plate and incubated at 37 °C in 5% CO_2_ and 100% humidity. At different designated sampling intervals (i.e., 1, 3, 7, 10 and 14 days), the amount of minocycline released into the supernatant was quantified using ultraviolet spectrophotometry (Shimadzu UV–vis Spectrophotometer UV-1603, Japan) at 348 nm (n = 3). A standard curve was used to determine the release profile of minocycline at different concentrations.

### Antibacterial assay

*S. gordonii* and *S. sanguis* were cultured separately in freshly prepared brain heart in fusion medium in an anaerobic chamber (N_2_: 80%, H_2_: 10%, CO_2_: 10%) at 37 °C. The antibacterial assay used bacterial concentrations of 5 × 10^5^ CFU/ml. All the Ti specimens tested were placed in 48-well plates. The wells were later filled with a bacterial suspension (1 ml per well), and then the samples were cultured at 37 °C in an anaerobic chamber incubator. After 24 h, the Ti samples were rinsed with PBS to eliminate non-adherent bacteria and were then subjected to ultrasonic treatment at 40 W for 5 min. Then, the suspensions were placed into new 48-well plates (1 ml per well). The number of viable planktonic bacteria adhering to the Ti samples was determined at different time points using the spread plate method. Antibacterial efficacy was assessed by CFU counting. A log transformation was used for this assay. All experiments were repeated three times.

CLSM (SP8, Germany) was utilized to characterize the viability of adherent bacteria on the samples. After culturing *S. gordonii* or *S. sanguis* on samples overnight in an anaerobic chamber at a concentration of 5 × 10^5^ CFU/ml, all the Ti specimens were gently rinsed twice with distilled water to remove unattached bacteria. Live and dead bacteria were identified via fluorescence staining with Live/Dead BacLight solution (Life Technologies Corporation, Carlsbad, CA), according to the manufacturer’s instructions. The CLSM data were used to generate 2D and 3D images.

### Cell culture

MC3T3-E1 cells were cultured in fresh Dulbecco’s modified Eagle’s medium (Gibco, Carlsbad, CA) containing 10% foetal bovine serum (Gibco) and 3% penicillin/streptomycin (Gibco) at 37 °C and 100% relative humidity in an incubator with an atmosphere consisting of 95% air and 5% CO_2_.

### LDH activity assay

LDH (Sigma-Aldrich) activity is representative of cytotoxicity; in this case, LDH activity represents the toxic effect of the samples on MC3T3-E1 cells. Three parallel groups of PTL-Ti and PTL-Ti-[HA/CS(MH)]_3_ samples were placed in 48-well plates. MC3T3-E1 cells were seeded at a density of 2 × 10^4^ cells/cm^2^ per well. After 24 h of incubation, the supernatants were sampled and centrifuged for the LDH activity assay. The optical density results were determined from absorbance values acquired at 450 nm, in accordance with the manufacturer’s instructions.

### MC3T3-E1 adhesion and proliferation assay

MC3T3-E1 cells were seeded on different samples in a 48-well plate at a density of 1.0 × 10^4^ cells/cm^2^ per well. To observe the cell morphology, after 6 and 24 h of culture, the samples were washed with PBS and fixed in 4% paraformaldehyde for 30 min at room temperature. The samples were washed five times with PBS. After being blocked with a bovine serum albumin for 1 h, the samples were washed three more times with PBS and stained with rhodamine phalloidin (Sigma-Aldrich) at room temperature in darkness for 50 min and further stained with DAPI (Sigma-Aldrich) for 3 min. The F-actin and cell nuclei were viewed by CLSM (SP8, Germany).

Cell proliferation on each sample was also measured using a CCK-8 assay after 0, 1, 2, 3, 4, 5 and 6 days of cell culture. All experiments were repeated three times.

### ALP activity

MC3T3-E1 cells were seeded onto each specimen in 48-well plates at a density of 2 × 10^4^ cells/cm^2^ per well. After sonication and centrifugation, aliquots of cell lysates were collected to assess ALP activity on the 7th and 14th days of culture. ALP levels were normalized to the total protein content, which was analysed using a Micro-BCA protein assay kit.

### Statistical analysis

All experiments were repeated at least three times to ensure the validity of observations, and the results are expressed as the mean ± standard deviation. The data were tested for homogeneity and then assessed statistically using one-way ANOVA and the least significant difference test. P < 0.05 was considered significant, and P < 0.01 was considered highly significant.

## Additional Information

**How to cite this article**: Guan, B. *et al*. Establishing Antibacterial Multilayer Films on the Surface of Direct Metal Laser Sintered Titanium Primed with Phase-Transited Lysozyme. *Sci. Rep*. **6**, 36408; doi: 10.1038/srep36408 (2016).

**Publisher’s note:** Springer Nature remains neutral with regard to jurisdictional claims in published maps and institutional affiliations.

## Figures and Tables

**Table 1:** Elemental composition of DMLS-Ti discs at different stages of surface functionalization with different treatments, as determined by XPS.

	C%	O%	N%	Ti%	Na%	Cl%	S%	Pi%	Al%	V%
DMLS-Ti	58.2 ± 0.2	27.7 ± 0.1	4.5 ± 0.1	8.6 ± 0.2	0	0	0	0	0.5 ± 0.1	0.4 ± 0.1
PTL-Ti	60.7 ± 0.3	19.8 ± 0.2	18.5 ± 0.1	0	0	0	0.6 ± 0.1	0.2 ± 0.1	0	0
PTL-Ti-HA	59.5 ± 0.2	26.4 ± 0.2	8.2 ± 0.2	0	3.6 ± 0.2	1.3 ± 0.2	0	0	0	0
PTL-Ti-HA/CS(MH)	62.7 ± 0.3	25.6 ± 0.3	9.4 ± 0.3	0	1.1 ± 0.2	0.9 ± 0.3	0	0	0	0
PTL-Ti-HA/CS(MH)]_3_	63.2 ± 0.2	24.9 ± 0.2	10.6 ± 0.2		0.7 ± 0.2	0.6 ± 0.2	0	0	0	0

**Table 2:** Mechanical properties of DMLS-Ti discs at different stages of surface functionalization with different treatments, as determined by nano-indentation.

	Modulus(GPa)	Hardness(GPa)
DMLS-Ti	71.2 ± 5.2	2.1 ± 0.4
PTL-Ti	57.9 ± 4.4	0.8 ± 0.2
PTL-Ti-HA	40.9 ± 3.1	0.6 ± 0.1
PTL-Ti-HA/CS(MH)]_3_	53.7 ± 3.8	0.7 ± 0.1
